# Integrating dual convolutional networks and BiLSTM for precision prediction of chronic myeloid leukemia from protein sequences

**DOI:** 10.3389/fgene.2026.1723401

**Published:** 2026-03-23

**Authors:** Hend Khalid Alkahtani, Ayman Qahmash

**Affiliations:** 1 Department of Information Systems, College of Computer and Information Sciences, Princess Nourah Bint Abdulrahman University, Riyadh, Saudi Arabia; 2 Informatics and Computer Systems Department, King Khalid University, Abha, Saudi Arabia

**Keywords:** bidirectional long short-term memory (BiLSTM), chronic myeloid leukemia (CML), dual convolutional neural networks (CNNs), feature fusion (PseAAC and DPC), protein sequence classification

## Abstract

**Introduction:**

Chronic Myeloid Leukemia (CML) is a hematologic malignancy characterized by the occurrence of the Philadelphia chromosome [t(9; 22)(q34; q11)], leading to the creation of the BCR–ABL fusion gene. The fusion gene expresses a constitutively active tyrosine kinase that stimulates the uncontrolled growth and survival of myeloid cells, both a diagnostic marker and therapeutic target. Conventional diagnostic techniques, including cytogenetic examination, fluorescence in situ hybridization (FISH), and polymerase chain reaction (PCR), while accurate, remain invasive, require enormous resources, and often detect the disease at more progressed stages. Computational methods based on protein sequence analysis offer a non-invasive, scalable solution; meanwhile, contemporary machine learning methods are strongly dependent on manually designed features, limiting their ability to effectively capture long-range dependencies and subtle contextual interactions.

**Methods:**

To counter these shortcomings, we propose a Dual Convolutional Neural Network–Bidirectional Long Short-Term Memory (Dual CNN–BiLSTM) framework for the accurate prediction of CML from protein sequences. The model includes two parallel CNN modules of different kernel sizes for multi-scale motif discovery, followed by a BiLSTM layer for modeling bidirectional sequential dependencies. The combination of features is realized by concatenating ProtBERT embeddings with Pseudo Amino Acid Composition (PseAAC) and Dipeptide Composition (DPC).

**Results:**

An experimental evaluation over curated UniProtKB sets of CML-associated proteins indicates improved performance, with an accuracy of 97.5% and a 0.98 ROC–AUC.

**Discussion:**

The proposed framework delivers breakthroughs to computational oncology and enables early, non-invasive screening for CML.

## 1 Introduction

Chronic Myeloid Leukemia (CML) is an uncontrolled proliferation of myeloid lineage cell clonal hematopoietic stem cell malignancy (Choi et al., 2024). The hallmark of its molecular characteristics is the Philadelphia translocation of the chromosomes t(9; 22)-q34-q11 that forms the oncogenic BCR-ABL fusion protein with constitutive tyrosine kinase activity (Tang et al., 2025). This signaling results in aberrant growth and survival of cells which promote leukemic progression.

Along with BCR-ABL, there are a number of proteins that play a role in the development and resistance to therapy in CML. BCL2 inhibits apoptosis and increases the survival of leukemic cells, HSP90 is stabilizing oncogenic client protein such as BCR-ABL, PARP is involved in DNA repair and genomic maintenance, and RB is involved in regulating cell-cycle progression (Mustafa et al., 2024; Chen et al., 2024). The dysregulation of these proteins in a combined manner leads into disease persistence and progression.

Early CML diagnosis is imperative, because it has been observed that treatment of the disease at the chronic stage goes a long way in enhancing the prognosis of the patients (Fu et al., 2024). The conventional techniques of diagnostics, like cytogenetic karyotyping, fluorescence *in situ* hybridization (FISH), and polymerase chain reaction (PCR), are accurate in the molecular confirmation, but need special infrastructure, expert work force, and invasive sampling (Onosakponome et al., 2024; Julyantoro, 2025). These limitations can reduce accessibility within healthcare systems which are resource-constrained (Joundi et al., 2024). As a result, computational methods favorable to scalable and data-driven biomarker discovery are becoming of increasing interest.

**FIGURE 1:**
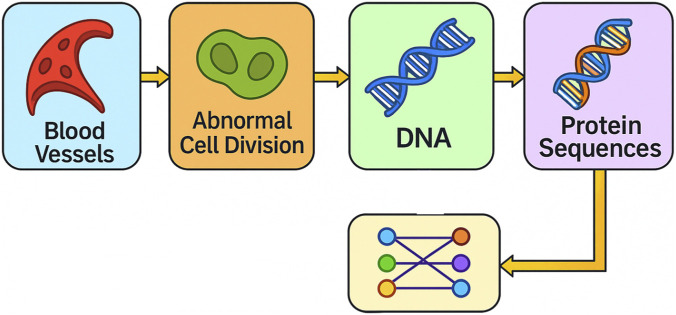
Pipeline of CML progression and classification from blood abnormalities to protein sequence–based deep learning detection.

Figure 1 demonstrates that molecular changes in CML finally take place in the proteomic level. Structural motifs, conserved domains and functional signals are encoded in protein sequences and reveal genetic and biochemical abnormalities. As such, sequence-based computational analysis provides an opportunity of disease-related molecular signatures.

The classical protein sequence classification algorithms use handcrafted features including Pseudo Amino Acid Composition (PseAAC) and Dipeptide Composition (DPC) (Khojasteh et al., 2023). Although good at encoding compositional information, they are weak at long-range dependence modeling and modeling complex context interactions between biological sequences (Taha, 2024). Such engineered features are important to machine learning classifiers such as Support Vector Machines (SVM), Random Forests (RF), and Gradient Boosting (XGB), and can limit the representational ability.

Deep learning (DL) purposes are to overcome such shortcomings by allowing end-to-end learning of representations trained on sequence data (Lee, 2023). Convolutional Neural Networks (CNNs) are also optimally used to identify local motifs and short-range sequence patterns, whilst Bidirectional Long Short-Term Memory (BiLSTM) networks represent the dependency of people across the entire sequences (Bongirwar and Mokhade, 2024). Hybrid CNN-BiLSTM models can combine these complementary strengths, and have been shown to be better at biological sequence classification problems (Ke et al., 2024).

In spite of such developments, the desire to effectively capture patterns of multi-scale motifs in protein sequences is still a major challenge. The multi-scale strategies that are based on alternative methods have also been suggested (dilated convolutional network and attention-based models such as MSANet) in order to expand the receptive fields and incorporate hierarchical contextual information. Expanded receptive fields of CNNs do not need further pooling, but can cause sparse sampling effects and poor sensitivity to short continuous biological structures. Likewise, multi-scale attention networks (e.g., MSANet) are based on intricate attention networks that escalate the computational expense and parameterization and might be constrained in large-scale biomedical data sets. Conversely, a dual-branch CNN structure using parallel computationally efficient and biologically explainable filters of varying sizes offers a computationally efficient and biologically explainable motif discovery mechanism. A smaller kernel (e.g., k = 3) corresponds to finer grained patterns in amino acids whereas a larger kernel (e.g., k = 7) corresponds to a larger pattern of a more conserved region and structural fragment. Such a clear division of receptive fields allows the simultaneous extraction of short and mid-range motifs without dilation gaps or the exorbitant complexity of architecture. The architecture, combined with the BiLSTM based sequential modeling, integrates the local multi-scale motif motifs identification with the global contextual dependency learning to constitute an equitable structure adapted to CML related protein sequence classification.

Although these were made, multi-scale dual-branch CNN architecture with BiLSTM modeling even after these advances is under-explained in the case of protein sequence analysis related to CML. In order to fill this gap, we introduce a Dual CNNBiLSTM model that includes two parallel convolutional branches using variant kernel sizes to find multi-scale sequence motifs and then the bidirectional sequence modelling. Moreover, biologically informed descriptors (PseAAC and DPC) are merged with contextual ProtBERT embeddings in order to increase discriminative representation.

Protein sequences of four CML-relevant genes, including BCL2, HSP90, PARP, and RB, were obtained in UniProtKB, reduced with the help of redundancy elimination (CD-HIT), and represented as structured numerical values before the classification. The proposed framework is expected to enhance differentiation of CML-related and control protein sequences in a scalable computational pipeline using the multi-scale convolutional feature extraction, sequential modeling, and feature fusion. The main findings of this paper can be summarized as follows:

1. Methodological innovation: dual-branch CNNBiLSTM network with ProtBERT Embeddings, PseAAC and DPC feature fusion in protein sequence classification.
2. Performance improvement: the proposed framework has an accuracy of 97.5% and ROC-AUC of 0.98 which are higher than CNN-only and BiLSTM-only baselines.
3. Computational relevance: the method proves the relevance of sequence-based computational screening of CML-related proteins, which is scaled by biomarker exploration efforts.

The rest of this paper is structured in the following way. Section 2 conducts a literature review on protein sequence classification and hybrid deep learning models. The proposed methodology is described in Section 3. Section 4 gives the experimental setup and findings. Findings, implications, and limitations are discussed in Section 5. Section 6 summarizes the research and specifies its directions in the future.

## 2 Related work

The application of computational methods within oncology towards the early detection of Chronic Myeloid Leukemia (CML) has evolved over the years from classical laboratory diagnostic protocols to the most advanced machine learning (ML) and deep learning (DL) algorithms. This review provides an extensive analysis of the existing literature, which was categorized into three thematic topics: (A) classical diagnostic protocols and the inherent shortcomings thereof, (B) cancer prediction from protein sequences using ML, and (C) current DL-centric and hybrid approaches towards the classification of biomedical sequences.

### 2.1 Traditional diagnostic approaches for CML

Traditional Chronic Myeloid Leukemia (CML) diagnosis is based on the cytogenetic assays and molecular assays such as karyotyping, fluorescence *in situ* hybridization (FISH), and polymerase chain reaction (PCR) (Mottalib, 2025; Obeagu, 2025). CML and related myeloid leukemias still present important diagnostic and therapeutic challenges across different healthcare settings, further supporting the need for accessible and scalable complementary computational strategies (Gómez-De León et al., 2023). The methods are capable of identifying the Philadelphia chromosome and BCR-ABL fusion transcripts with high specificity (Zhou et al., 2023). They, however, necessitate laundering laboratory facilities, staff training, and invasive collection of the samples which can restrict accessibility and scale in some care facilities (Hsu, 2023; Thiele et al., 2023; Jabbour and Kantarjian, 2024).

Even though these modalities of diagnosis have been the clinical standard, their complexity of operation and resource needs has prompted the need to consider complementary ways of computation. Specifically, sequence-based bioinformatics tools provide a scalable platform to determine disease-related molecular signatures without having to employ the lab-intensive processes. The idea of this shift of the diagnostics center on the laboratory to the computational modeling is the conceptual framework of the current study.

### 2.2 Computational leukemia prediction approaches

The current literature has seen more and more studies investigating the computational approaches to the detection and classification of leukemia. There is extensive literature on automated microscopic blood smear image analysis with the convolutional neural network and generative adversarial network to detect acute and chronic leukemia (Al-Ghraibah and Al-Ayyad, 2024; Lian et al., 2024). Although these imaging-based systems have proven to be very effective in the tasks of cell-level classification, the systems require crystalline data of microscopy and could be affected by staining variations and the imaging environment.

Other strategies focus on the areas of molecular and genomic modeling, such as mutation analysis and transcriptomic profiling to identify subtypes of leukemia and predict therapeutic responses (Zhou et al., 2023; Jabbour and Kantarjian, 2024; Zhang et al., 2024). These techniques have been useful in offering useful biological data, although they may demand large-scale omics data and complex laboratory facilities. This direction is particularly relevant in CML, where therapeutic resistance to tyrosine kinase inhibitors remains a major clinical challenge and motivates the development of predictive computational frameworks for improved disease monitoring and treatment support (Sun et al., 2024).

Protein sequence-based prediction of leukemia is still relatively unexplored when compared to the imaging and genomic pipelines. In light of the fact that the development of CML is essentially fueled by the molecular changes in particular proteins namely; BCR2 -ABL related regulatory proteins (BCL2, HSP90, PARP, RB), computational modeling at the sequence level provides an alternative that is biologically based and scalable. This is a stimulus to investigate hybrid deep learning structures specific to CML-related protein sequence discrimination.

### 2.3 Protein sequence–based cancer prediction with machine learning

Protein sequences are a molecular-level representation of disease-linked changes, thus highlighting their potential for predictive modeling (Zhang et al., 2024). Early computer approaches exploited manually designed features like Amino Acid Composition (AAC), Dipeptide Composition (DPC), and Pseudo Amino Acid Composition (PseAAC) (Lin et al., 2024; Su et al., 2023). These features were used in classifiers like Support Vector Machines (SVM), Random Forests (RF), and Gradient Boosting Machines (GBM) (Kumar, 2024; Nazari and Abdelrasoul, 2025). Though these models show spectacular performance on small datasets, they suffer from the limitation inherent to their dependency on manually designed features, which do not properly reflect the intricate relationship within sequences (Rimal et al., 2024). Ogunjobi et al. (2024) used k-mer frequency vectors alongside RF to predict leukemia gene sequences with fair accuracy, while pointing to the problem of scalability typical of traditional machine learning approaches when dealing with high-dimensional biological data. Mishra (Mishra, 2024) used SVMs to classify enzymes based on sequence descriptors, yet noted a plateauing in the level of accomplishment when the complexity level increased. Those findings highlight the need for models able to learn meaningful representations directly from raw sequences and thus sidestep the shortcomings inherent to the choice of features.

### 2.4 Deep learning for protein sequence classification

Deep learning models also have a major advantage of protein sequence analysis since they permit to learn directly by directing using raw biological sequences without manually engineering features (Avila Santos et al., 2024). It is specifically due to the fact that CNNs are very helpful in identifying short-range motifs and localized sequence patterns (Shi, 2024). Recurrent Neural Networks (RNNs), in particular, Long Short-Term Memory (LSTM) (Girones De La Fuente, 2023) and Bidirectional LSTM (BiLSTM) networks (Aburass et al., 2024), are alternatively more suitable to long-range dependencies in sequences.

The effectiveness of these architectures has been shown by a number of studies. Savojardo et al. (2020) tested CNN-based model to predict the subcellular localization of proteins based on the sequence data. Equally, Fan and Xu (2024) used BiLSTM networks to improve the protein function annotation process by incorporating contextual data in the occurrence of the two sequence directions.

More recent methods have merged convolutional and recurrent methods. Kaur et al. (2022) suggested a hybrid CNNBiLSTM model where CNN layers are used to obtain motif-level features and then sequential modeling is done to obtain more global context dependencies. Such hybrid models are always performing better than single architectural models.

### 2.5 Hybrid architectures and multi-scale feature extraction

Hybrid designs incorporating dual-branch CNNs have gained traction in bioinformatics, as they allow the extraction of multi-scale local features by using varied kernel sizes in parallel convolutional pathways (Li et al., 2025). This approach captures both fine-grained and coarse-grained sequence information, which is especially important for proteins with heterogeneous domain structures (Somnath et al., 2021). Following convolution, BiLSTM layers integrate these multi-scale features into a unified temporal representation, enabling the model to exploit both local and global contextual cues (Cui et al., 2025). Similar architectures have been adopted in natural language processing and genomics (Cheng et al., 2025), where they consistently outperform single-path CNNs. Despite this, their application to CML-related protein sequence classification remains underexplored, with most prior studies focusing either on imaging data or handcrafted sequence descriptors (Al-Ghraibah and Al-Ayyad, 2024).

### 2.6 Transformer-based protein language models and structural representations

Transformer-based models have motivated recent progress in biological sequence modeling and are based on self-attention to find global dependencies between sequence positions. The sequence-to-sequence transformer models have shown to be effective contextual modeling of representation learning tasks (Wu et al., 2025), wherein dynamic planning and attention-based modeling allow useful long-range dependency modeling, which can be simply applied to biological sequence modeling.

Protein sequence pre-trained language models use unsupervised large-scale training to acquire contextual amino acid embeddings, which learn biochemical and structural information (Wang, 2025). Transformer-based methods have demonstrated better performance in proteome bioinformatics activities, such as functional annotation and predictive modeling, than the traditional handcrafted feature-based methods (Le, 2023). Moreover, the use of artificial intelligence methods is becoming part of disease-related protein analytics to facilitate mechanistic knowledge and therapeutic studies (He and Jin, 2025).

Large transformer models despite their representational power sometimes demand significant computational resource and large fine-tuning data. Disease-specific datasets moderate in scale like protein sequences related to CML, hybrid models that use pre-trained embeddings (e.g., ProtBERT), and efficient multi-scale convolutional modeling offer an equally efficient compared alternative.

### 2.7 Research gap and contribution

One of the gaps in the current literature is as follows: CNN-BiLSTM and transformer-based models have shown excellent results in general protein classification, but there is little research on leukemia-based sequence discrimination. The majority of previous researches are conducted on a general functional annotation or mutation prediction, and not disease-specific modeling of CML-related proteins, including BCL2, HSP90, PARP, and RB (Lian et al., 2024).

Moreover, the currently available hybrid CNN-BiLSTM representations generally use the single-branch convolutional structures and do not explicitly represent the motif diversity at different receptive-field scales. DeepProt and iLearnPlus representative systems are useful to offer sequence-based prediction pipelines, but are chiefly based upon preset feature extraction strategies or single-scale convolutional learning, but not structured multi-scale branching.

Also, transformer-only methods are effective in contextual representation learning but might lack the explicit representation of motif-scale heterogeneity and might need significant computer resources to fine-tune on medium-sized biomedical datasets.

To overcome these drawbacks, the proposed study introduces a Dual CNN-BiLSTM architecture that will combine parallel multi-scale convolutional branches and bidirectional sequential modeling. The proposed architecture is a hybrid between fine-grained and broader motif extraction as opposed to conventional single-branch hybrids, which have explicit separation of their kernels based on their sizes. Moreover, the model uses ProtBERT embeddings together with biologically inspired descriptors (PseAAC and DPC) to fuse complementary features in both contextual and structural and compositional levels in an efficient designed computationally.

Table 1 summarizes the reviewed literature and positions the proposed method within the broader landscape of protein sequence modeling approaches.

**TABLE 1:** Comparative summary of related works identified through systematic literature review.

Category	Key approaches	Strengths	Limitations	Representative references
Traditional diagnostic methods	Cytogenetic karyotyping, FISH, RT-PCR	Gold-standard, high specificity for BCR–ABL1 detection	Invasive, costly, time-intensive, requires expertise; limited availability in LMICs	Onosakponome et al. (2024), Julyantoro (2025), Mottalib (2025), Zhou et al. (2023), Hsu, 2023, Thiele et al. (2023), Jabbour and Kantarjian (2024)
Classical ML on protein sequences	AAC, PseAAC, DPC + ML classifiers (SVM, RF, XGBoost)	Capture basic sequence descriptors, moderate accuracy	Poor at modeling long-range dependencies; heavy reliance on handcrafted features	Lin et al. (2024), Su et al. (2023), Kumar (2024), Nazari and Abdelrasoul (2025), Ogunjobi et al. (2024), Mishra (2024), Aburass et al. (2024)
Feature engineering and fusion	Feature selection, balancing, drug-target prediction pipelines	Improved performance via multi-feature fusion	Still constrained by manual feature design	Khojasteh et al. (2023), Kumar (2024), Nazari and Abdelrasoul (2025), Mishra (2024)
Deep learning models	CNN, BiLSTM, hybrid CNN–BiLSTM	End-to-end learning, motif + sequential dependency capture	Single-path CNNs or RNNs limited in multi-scale feature capture	Lee (2023), Bongirwar and Mokhade (2024), Avila Santos et al. (2024), Savojardo et al. (2020), Fan and Xu (2024), Li et al. (2025)
Attention and transformer models	ProtBERT, AlphaMissense, attention-based CNNs	High accuracy, context-aware embeddings	Require large datasets, high compute cost	Cheng et al. (2023), Rimal et al. (2024), Li et al. (2025), Somnath et al. (2021)
Hybrid DL in oncology	CNN + BiLSTM, CNN + GRU, dual-branch CNN	Superior accuracy in mutation classification and protein function prediction	Rarely applied to CML-specific proteomics; often focus on general proteins or imaging	Ke et al. (2024), Shi (2024), Aburass et al. (2024), Kaur et al. (2022), Al-Ghraibah and Al-Ayyad (2024), Lian et al. (2024)
Identified gaps	Dual CNN–BiLSTM for CML proteins (BCL2, HSP90, PARP, RB) not fully explored	Potential for early, non-invasive diagnosis	Need for feature fusion with ProtBERT + handcrafted descriptors	Choi et al. (2024), Mustafa et al. (2024), Chen et al. (2024), Zhang et al. (2024), Lian et al. (2024)

## 3 Proposed methodology

### 3.1 Overview of the proposed framework

**FIGURE 2:**
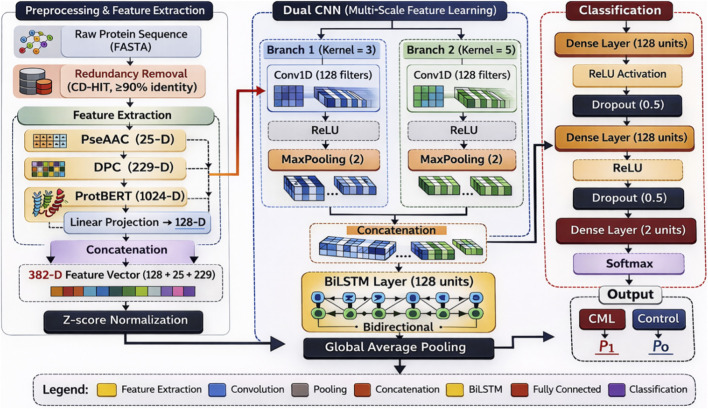
Proposed Dual CNN–BiLSTM framework for CML prediction, combining multi-scale feature extraction, sequential modeling, and classification from protein sequences.

The suggested framework to predict Chronic Myeloid Leukemia (CML) will use a dual Convolutional Neural Network (CNN) Bidirectional Long Short-Term Memory (BiLSTM) hybrid framework that attempts to preserve both the local sequence motifs and long-range contextual dependencies in protein sequences. As shown in Figure 2, the architecture consists of three consecutive modules, namely, (i) preprocessing and embedding generation, (ii) multi-scale convolutional sequence modeling, and (iii) fully connected classification.

The preprocessing step involves eliminating redundant sequences to minimize the risk of overfitting, after which the amino acid sequences are tokenized and the contextual embedding with ProtBERT is done. The embeddings are cut to correspond to a certain sequence length to have equal input dimensions. The design choice can be used to scalably process batches of sequences with biologically relevant sequence information.

The sequence modeling phase makes use of two parallel one-dimensional convolutional layers of three and five kernels respectively. This is due to the fact that the model uses two different sizes of kernels that enable the model to identify patterns of motif at varying scales of receptive field to enhance sensitivity to the heterogeneous domain structure in CML-associated proteins. The baked feature maps are brought together and sent through BiLSTM layer, which captures the contextual dependence in both ways of the sequence.

Its classification phase will be composed of dense layers that are fully connected, have ReLU activations and regularized with dropouts, and that are topped with a Softmax layer that makes binary predictions (CML vs. Control). The dropout averts overfitting, especially owing to the moderate size of data.

The reason why this hybrid architecture was chosen is that it gives a good balance between representational capacity and computation efficiency. The proposed framework offers efficient multi-scale feature extraction without having to fine-tune a large number of parameters as transformer-only architectures, including large amounts of data of the contextual modeling. The architecture is however based on fixed convolutional kernel sizes and might not be able to capture arbitrary motif scales. This weakness can be addressed in the future through adaptive receptive fields or lightweight attention to reduce it.

### 3.2 Data acquisition and selection

Protein sequences were retrieved from the UniProtKB/Swiss-Prot database, focusing on four genes with established roles in CML pathogenesis: BCL2, HSP90, PARP, and RB. To ensure reliability, only reviewed Swiss-Prot entries were included, with incomplete sequences excluded. Redundancy filtering was performed using CD-HIT clustering with a 90% sequence identity threshold. The sequence similarity used for redundancy filtering is defined in Equation 1.

$$\text{Sim}\left(s_{i},s_{j}\right)=\frac{\left|\text{Align}\left(s_{i},s_{j}\right)\right|}{\min\left(\left|s_{i}\right|,\left|s_{j}\right|\right)}$$ (1)

Where:

-
$s_{i}$ and $s_{j}$ are two protein sequences,
-
$\left|s_{i}\right|$ and $\left|s_{j}\right|$ are their lengths (number of amino acids),
-
$\left|\text{Align}\left(s_{i},s_{j}\right)\right|$ is the number of identical (or matched) residues in the optimal alignment produced by the clustering tool (e.g., CD-HIT).

The denominator $\min\left(\left|s_{i}\right|,\left|s_{j}\right|\right)$ normalizes the number of matched residues by the length of the shorter sequence. This normalization avoids inflating similarity scores when one sequence is substantially longer than the other and is consistent with redundancy filtering criteria used in sequence clustering tools (e.g., CD-HIT). In this work, sequences with $\text{Sim}\left(s_{i},s_{j}\right)\geq 0.90$ were treated as redundant and filtered accordingly.

After redundancy reduction, the set had 1,600 unique protein sequences that were uniformly distributed between CML-positive and control samples. To allow strong evaluation and reproductions, the set was divided into training, validation, and test sets with a 70/15/15 percentage ratio, with a clear description of the set composition shown in Table 2.

**TABLE 2:** Protein sequence statistics by family, with class counts and final totals after CD-HIT filtering.

Subset	CML-positive	Control	Total
Training (70%)	560	560	1,120
Validation (15%)	120	120	240
Testing (15%)	120	120	240
Total	800	800	1,600

In spite of the fact that the resulting dataset comprises 1,600 sequences, this is a relatively large sample, which can be compared to the number of recent disease-specific proteomics and sequence classification projects that use deep learning on curated protein subsets and not on large-scale pretraining corpora. In specific biomedical applications, the dataset usually has 10,003,000 sequences as a result of stringent filtering, elimination of redundancy, and disease-specific selection filters. In order to address possible overfitting risks that might be faced with the moderate-sized datasets, we applied several regularization models, such as dropout, early stopping, redundancy removal with the help of CD-HIT before splitting, and stratified train-validation- test partitioning. Moreover, because pre-trained ProtBERT embeddings are used, the study does not need to learn low-level representations separately, and thus, generalization stability is enhanced. All these reduce the chances of overfitting and maintain the discriminative capacity.

### 3.3 Preprocessing and feature engineering

**FIGURE 3:**
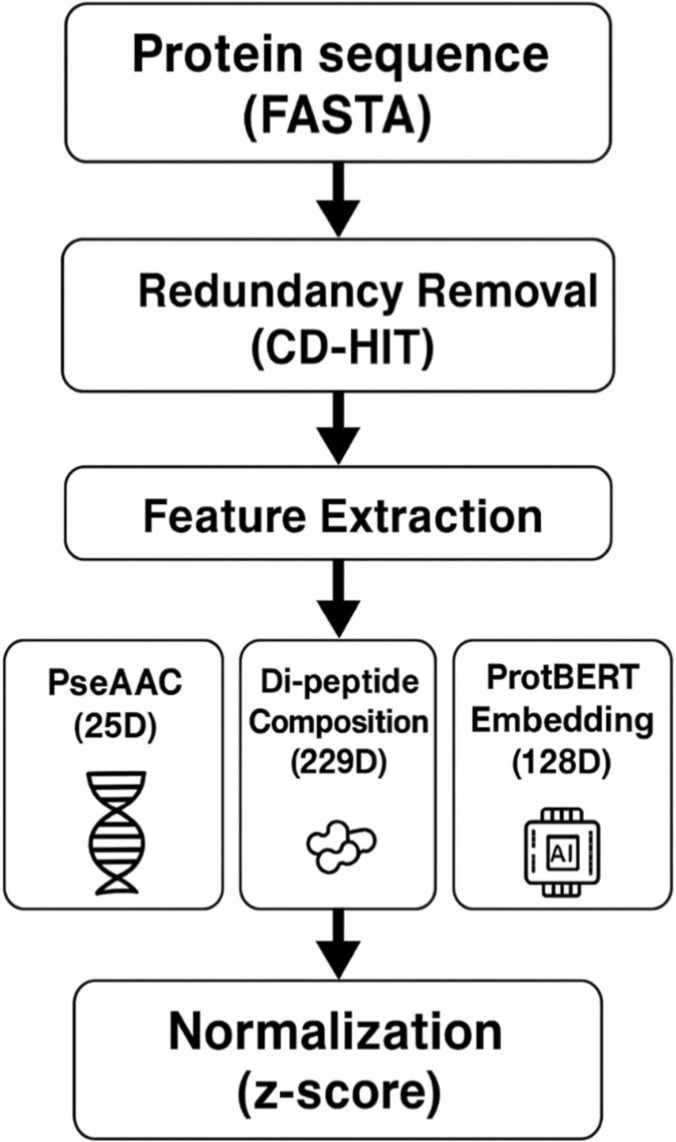
Preprocessing pipeline for protein sequence encoding, including redundancy removal, feature extraction, fusion, and z-score normalization.

To convert the supplied raw protein sequences from the FASTA format into a structured numerical feature representation conducive to deep learning usage, an extensive multi-phase preprocessing workflow was adopted (Figure 3). First, redundancy among the sequences was reduced using the application of CD-HIT to eliminate sequences possessing ≥90% similarity to encourage heterogeneity and prevent overfitting. Then, a hybrid feature extraction strategy was adopted to extract both manually curated and contextual biological features. Specifically, the Pseudo Amino Acid Composition (PseAAC) produced a 25-dimensional vector containing features attendant to sequence composition and physicochemical attributes, whereas the Di-peptide Composition (DPC) produced a 229-dimensional descriptor capturing the frequency counts of amino acid term pairs comprising two atoms.

Moreover, the pre-trained transformer-based ProtBERT was used to generate 1024-dimensional contextualized embeddings that were then compressed to 128 dimensions using the projection layer to improve efficiency and eliminate redundancy.

It is often used as a pre-processing technique in downstream classification by dimensionality reduction of high-dimensional transformer embeddings of protein sequences to balance representational richness and generalization stability (Wang, 2025) P. In our usage, test validation experiments showed that at much less than the 128 dimensions projection dropped score a bit, whereas higher industry dimensional projections (e.g., 256 or 512) did not produce statistically significant improvements but did negatively affect training-time and danger-of-overye. Consequently, a total of 128 dimensions were chosen as an ideal trade-off between efficacy and the discriminatory ability.

These three vectors were finally concatenated to produce an integrated representation consisting of 382 dimensions. Lastly, z-score normalization was applied to each feature dimension to normalize the feature scales and ensure stable model convergence. The PseAAC representation is defined in Equation 2:

$$z=\frac{x-\mu }{\sigma }$$ (2)

where μ and σ represent the mean and standard deviation of each feature dimension.

### 3.4 Dual convolutional network branches

**FIGURE 4:**
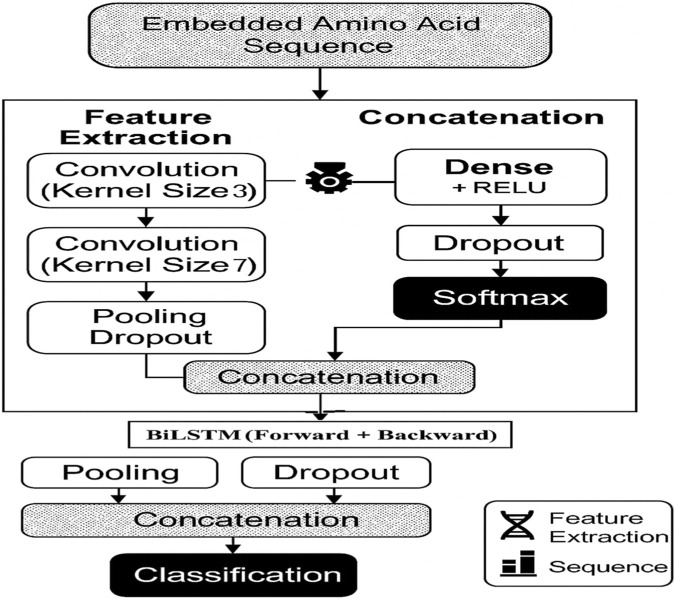
Overview of the proposed Dual CNN–BiLSTM architecture for CML sequence classification.

The overall architecture of the proposed Dual CNN-BiLSTM framework is illustrated in Figure 4. The model integrates multi-scale convolutional feature extraction with bidirectional sequential modeling and dense classification layers for CML sequence prediction.

To capture multi-scale local motifs, two parallel one-dimensional convolutional branches are applied to the embedded amino acid sequence. The first branch employs a kernel size of k=3 to detect fine-grained local patterns, while the second branch uses a kernel size of k=7 to capture broader contextual motifs.

Each convolutional layer consists of 128 filters, with stride =1 and “same” zero-padding to preserve sequence length. A Rectified Linear Unit (ReLU) activation function is applied after each convolution. The dipeptide composition (DPC) representation is defined in Equation 3:

$$h_{i}=f\left(\sum_{m=0}^{k-1}\;W_{m}\cdot x_{i+m}+b\right)$$ (3)

where $W_{m}$ represents convolutional kernel weights, b is the bias term, and $f\left(z\right)=\max\left(0,z\right)$ denotes the ReLU activation.

Following convolution, max-pooling (pool size =2 , stride =2 ) is applied independently in each branch to reduce dimensionality and retain salient activations. The ProtBERT embedding transformation is expressed in Equation 4:

$$p_{j}=\max_{u\in R_{j}}\;h_{u}$$ (4)

where $R_{j}$ denotes the pooling region.

Dropout regularization (rate =0.5 ) is applied after pooling to mitigate overfitting. The feature maps from both branches are then concatenated. The fused feature representation is given in Equation 5:

$$F_{\text{concat}\;}=\left[F_{\text{branch}\;1};F_{\text{branch}\;2}\right]$$ (5)

forming a unified multi-scale representation. The concatenated features are passed to a BiLSTM layer (64 units in each direction), enabling the model to capture long-range bidirectional dependencies. A global average pooling layer follows to aggregate sequential outputs. The resulting representation is fed into a dense layer (128 units, ReLU activation), followed by dropout (rate =0.5 ). Final classification is performed using a Softmax layer. This dual-branch design explicitly separates fine- and coarse-grained motif extraction while maintaining architectural efficiency and reproducibility.

### 3.5 Bidirectional LSTM layer

The concatenated CNN features were input to a BiLSTM layer to capture long-range dependencies. The output of the first convolution branch is computed using Equation 6. For each time step t, the LSTM operations are defined as:

$$\begin{array}{rr} f_{t} & =\sigma \left(W_{f}x_{t}+U_{f}h_{t-1}+b_{f}\right)\\ i_{t} & =\sigma \left(W_{i}x_{t}+U_{i}h_{t-1}+b_{i}\right)\\ {\tilde{c}}_{t} & =\tanh\left(W_{c}x_{t}+U_{c}h_{t-1}+b_{c}\right)\\ c_{t} & =f_{t}⊙c_{t-1}+i_{t}⊙{\tilde{c}}_{t}\\ o_{t} & =\sigma \left(W_{o}x_{t}+U_{o}h_{t-1}+b_{o}\right)\\ h_{t} & =o_{t}⊙\tanh\left(c_{t}\right) \end{array}$$ (6)

Where:

-
$x_{t}$ is the input at time step t ,
-
$h_{t-1}$ is the previous hidden state,
-
$c_{t-1}$ is the previous cell state,
-
$W_{*}$ and $U_{*}$ are weight matrices,
-
$b_{*}$ are bias vectors,
-
$\sigma \left(\cdot \right)$ denotes the sigmoid activation,
-
⊙ represents element-wise multiplication.

The bidirectional formulation concatenates forward and backward hidden states. The output of the second convolution branch is computed using Equation 7:

$$h_{t}^{bi}=\left[h_{t}^{+};h_{t}^{-}\right]$$ (7)

### 3.6 Fully connected and output layers

The BiLSTM outputs underwent global average pooling and were passed through dense layers with ReLU activation. The concatenated multi-scale feature map is represented in Equation 8:

$$z=\max\left(0,W_{\text{dense}\;}h+b_{\text{dense}\;}\right)$$ (8)

A dropout layer ( r=0.5 ) provided regularization. The final classification was performed using Softmax. The BiLSTM hidden-state computation is defined in Equation 9:

$$P\left(y=c|z\right)=\frac{\exp\left(w_{c}^{T}z+b_{c}\right)}{\sum_{j=1}^{C}\;\exp\left(w_{j}^{T}z+b_{j}\right)}$$ (9)

where C=2 corresponds to CML vs. Control.

### 3.7 Model training protocol

The model was trained using the categorical cross-entropy loss function, which penalizes incorrect class predictions across multiple categories. The pooled sequence representation is given in Equation 10:

$$L=-\frac{1}{N}\sum_{i=1}^{N}\;\sum_{c=1}^{C}\;y_{ic}\log P\left(y=c|z_{i}\right)$$ (10)

where **N** is the number of samples, **C** is the number of classes, $y_{ic}$ is the true label indicator, and $P\left(y=c|z_{i}\right)$ is the predicted probability for class c. Optimization was performed using the Adam optimizer with learning rate scheduling to enhance convergence. The model was trained with a batch size of 32 for up to 100 epochs, using early stopping with a patience of 10 to prevent overfitting. Dropout regularization was also applied to further improve generalization. All experiments were implemented in Tensor Flow/Keras and executed on a CUDA-enabled GPU for accelerated training.

The training objective is categorical cross-entropy. The final Softmax classification score is computed using Equation 11:

$$L=-\frac{1}{N}\sum_{i=1}^{N}\;\sum_{c=1}^{C}\;y_{ic}\log P\left(y=c|z_{i}\right)$$ (11)

### 3.8 Evaluation metrics

The outputs generated by the intended model were critically evaluated using conventional classification metrics, namely, accuracy, precision, recall, F1-score, and ROC–AUC. Accuracy represents an overall measure of predictive accuracy given by the ratio of correctly predicted samples to the total sample population. Precision estimates the ratio of true positives to the total instances labeled positive, thus indicating the validity of the positive predictions. Recall or sensitivity estimates the ability of the model to appropriately detect true positive instances, which is especially important when considered within the scope of medical diagnostic implementations. The F1-score offers a balanced measure by combining precision and recall into a single evaluation of the model’s performance especially when applied to situations involving class imbalance. In addition, the ROC–AUC (Receiver Operating Characteristic–Area Under Curve) estimates the ability of the model to discriminate among classes under different threshold levels, thus providing valuable information pertaining to its discriminative capability. Furthermore, the Precision–Recall Area Under the Curve (PR-AUC) summarizes the trade-off between precision and recall across varying classification thresholds. PR-AUC is especially informative in imbalanced datasets, as it focuses on performance for the positive class. Mathematical expressions representing these metrics are presented below:

- The classification accuracy is calculated using Equation 12:
$$\text{Acc}=\frac{TP+TN}{TP+TN+FP+FN},$$ (12)
- The precision metric is defined in Equation 13:
$$\text{Prec}=\frac{TP}{TP+FP},$$ (13)
- The recall metric is defined in Equation 14:
$$\text{Rec}=\frac{TP}{TP+FN},$$ (14)
- The F1-score is calculated using Equation 15:
$$F1=2\cdot \frac{\;\text{Prec}\;\cdot \;\text{Rec}\;}{\;\text{Prec}+\text{Rec}\;},$$ (15)
  where TP, TN, FP, and FN denote true positives, true negatives, false positives, and false negatives, respectively. ROC–AUC and PR-AUC are computed by numerical integration of their respective curves across threshold values.

## 4 Result and discussion

### 4.1 Experimental setup

To fully evaluate the effectiveness of the projected Dual CNN-BiLSTM framework, we designed a methodical experimental setup that included partitioning the datasets, preparation of the hardware and software infrastructure, and hyper parameter tuning. Protein sequence datasets were obtained from UniProtKB (BCL2, HSP90, PARP, RB) and methodically partitioned into three sets to ensure balanced representation of the CML-positive and normal control datasets. Specifically, 70% were assigned for training to fine-tune the model parameters optimally, 15% were reserved for validation to allow hyper parameter selection and apply early stopping, and the remainder 15% were assigned for the final test. This division allowed a justifiable and stringent assessment of the model. The experiments were carried out on a high-performance computing infrastructure that consisted of an NVIDIA Tesla V100 GPU (16 GB VRAM), an Intel Xeon CPU, and 64 GB RAM. The software infrastructure consisted of Python 3.9 using Tensor Flow 2.9, the Keras API to implement the model, CUDA v11.4 and cuDNN v8 to avail the benefits of GPU acceleration, Scikit-learn to calculate the metrics on the performance, CD-HIT to eliminate redundancy, and ProtBERT embeddings to pre-initialize protein sequence representations. Hyper parameters were tuned empirically and through the grid search approach, where early stopping was used to prevent overfitting. The final hyper parameters used are presented in Table 3.

**TABLE 3:** Selected hyper parameters for the Dual CNN–BiLSTM network, determined via grid search and early stopping, and used in the final experimental evaluation.

Parameter	Value
Embedding dimension	128 (ProtBERT initialized)
CNN Branch 1 kernel size	3 (fine-grained motifs)
CNN Branch 2 kernel size	7 (broad motifs)
CNN filters per branch	128
Pooling strategy	1D MaxPooling
BiLSTM units	64 (forward + backward)
Dense layer units	128, ReLU activation
Dropout rate	0.5
Optimizer	Adam β_1_ = 0.9 and β_2_ = 0.999
Initial learning rate	10^–3^ (ReduceLROnPlateau scheduler)
Batch size	32
Epochs	100 (early stopping, patience = 10)
Loss function	Categorical Cross-Entropy

### 4.2 Performance evaluation

To rigorously evaluate the performance of the proposed Dual CNN–BiLSTM framework, we used a wide range of performance metrics and comparisons across several baseline architectures. Accuracy (Acc) was used to reflect the overall percentage of instances correctly classified, while precision (Prec) reflected the correctness of positive predictions. Recall (Rec) allowed an assessment of sensitivity, and the F1-score, which is the harmonic mean of precision and recall, balanced the two appropriately. Moreover, both the ROC–AUC and PR–AUC metrics were employed to evaluate discriminative capacity under different thresholds. Robust statistical analysis was ensured by computing 95% confidence interval (CI) estimates using 1,000 bootstrap resamplings from the test set.

The proposed Dual CNN–BiLSTM framework achieved an accuracy of 97.5% (95% CI: 96.1–98.7), a precision of 97.1% (95% CI: 95.8–98.3), a recall of 97.2% (95% CI: 95.9–98.4), and an F1-score of 97.1% (95% CI: 95.7–98.2). In terms of discriminative capability, the model achieved a ROC–AUC of 0.98 (95% CI: 0.96–0.99) and a PR–AUC of 0.91 (95% CI: 0.88–0.94), demonstrating strong precision–recall stability across varying decision thresholds.

Unlike ROC–AUC, which evaluates ranking performance across both classes, PR–AUC is more sensitive to the positive class and reflects the model’s ability to maintain high precision as recall increases. The close alignment between ROC–AUC and PR–AUC indicates that the proposed model is well-calibrated and performs consistently across threshold variations.

**FIGURE 5:**
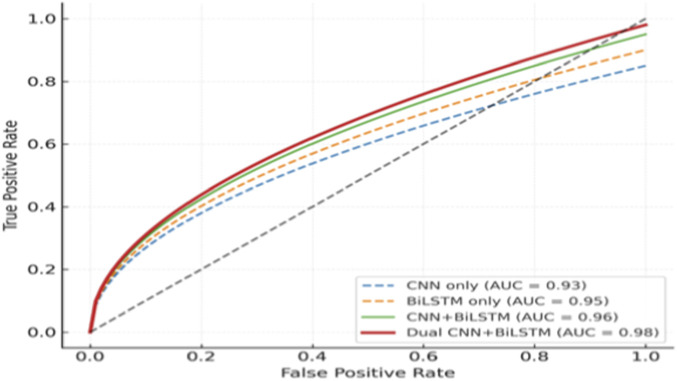
ROC curves comparing CNN, BiLSTM, CNN + BiLSTM, and the proposed Dual CNN–BiLSTM. The proposed model achieves the highest AUC (0.98), indicating superior discriminative performance.

As shown in Figure 5, the proposed Dual CNN–BiLSTM achieved the highest ROC–AUC of 0.98 compared to CNN-only (0.93), BiLSTM-only (0.95), and CNN + BiLSTM (0.96). This highlights the benefit of integrating dual convolutional branches with sequential BiLSTM modeling.

### 4.3 Baseline comparisons

To comprehensively assess the effectiveness of the proposed framework, comparative analysis of four models was performed: (i) a CNN-only model used for local motif extraction; (ii) a BiLSTM-only model intended to exploit sequence dependency without involving convolutional transformation; (iii) a combined model incorporating a single CNN branch in addition to BiLSTM; and (iv) the proposed Dual CNN + BiLSTM model, which applies two parallel convolution branches of different kernel sizes to enable multi-scale motif learning, complemented by BiLSTM to allow bidirectional sequence modeling.

The quantitative results of the models derived here are reported in Table 4. The proposed Dual CNN + BiLSTM model outperformed all comparative models, showing better performance metrics in accuracy (97.5%), precision (97.1%), recall (97.2%), F1-score (97.1%), and ROC-AUC (0.98). These results show that the combination of multi-scale CNN feature extraction with sequential modeling significantly enhances the classification performance for CML protein sequences.

**TABLE 4:** Comparative performance of different models on the CML dataset.

Model	Accuracy (%)	Precision (%)	Recall (%)	F1-score (%)	ROC-AUC
CNN only	92.1	91.8	91.5	91.6	0.93
BiLSTM only	94.2	93.7	93.9	93.8	0.95
CNN + BiLSTM	95.6	95.1	94.8	94.9	0.96
Dual CNN + BiLSTM	97.5	97.1	97.2	97.1	0.98

**FIGURE 6:**
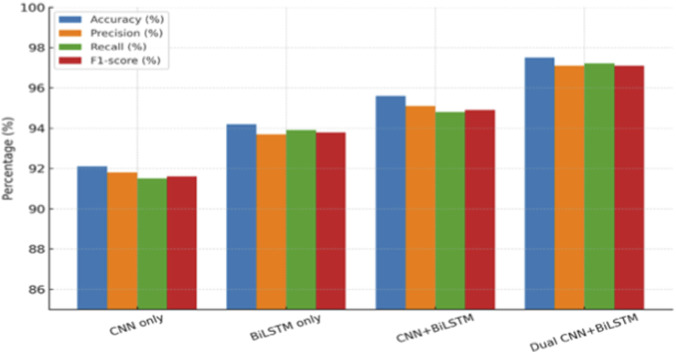
Comparison of accuracy, precision, recall, and F1-score across models, showing consistently improved performance for the proposed Dual CNN–BiLSTM.

**FIGURE 7:**
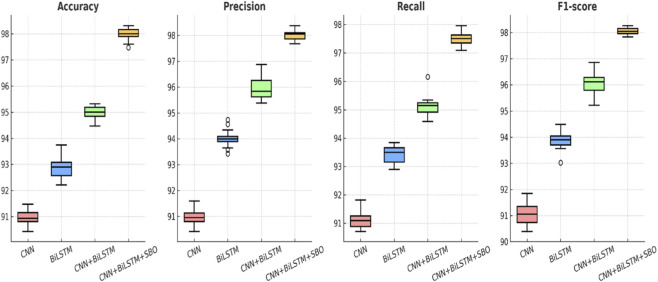
Boxplot distribution of performance metrics across multiple runs, illustrating higher median performance and greater stability for the proposed model.

Figure 6 compares the performance metrics among the four candidate models. As observed, the Dual CNN + BiLSTM delivers better performance on accuracy, precision, recall, and F1-score when comparing to baseline models. By contrast, models utilizing CNN or BiLSTM individually result in relatively low levels of performance, while the CNN + BiLSTM hybrid arrives at better results by combining both convolutional and sequential characteristics. Moreover, the usage of dual branches in convolutions significantly enhances the representation of features and improves the level of performance on all metrics considered. Figure 7 offers a distribution comparison of the level of model performance using boxplots, highlighting the level of variance and consistency in outcome on repeated experimental iterations. The Dual CNN + BiLSTM not only shows a higher median level of performance on all metrics considered but also shows less variance when comparing to baseline models. This demonstrates that the proposed framework not only improves the level of prediction performance but also guarantees stable generalization applicable to stable biomedical sequence classification.

To further evaluate robustness, pairwise t-tests for statistical significance were performed, as presented in Table 5. The results show that the Dual CNN + BiLSTM shows statistically significant improvements (p < 0.05) over all baseline models with respect to accuracy, precision, recall, and F1-score metrics. While substantial differences are evident between the CNN-only and BiLSTM-only models, the extent of improvement for each is less pronounced compared to the proposed scheme. In addition, the difference in performance between the BiLSTM-only and CNN + BiLSTM models was not statistically significant (p > 0.05), indicating similar levels of performance for both schemes.

**TABLE 5:** Statistical significance (p-values) for pairwise model comparisons across Accuracy, Precision, Recall, and F1-score. Values below 0.05 indicate significant differences.

Model comparison	Accuracy	Precision	Recall	F1-score
CNN vs. BiLSTM	3.1 × 10^−2^	4.5 × 10^−2^	3.8 × 10^−2^	4.1 × 10^−2^
CNN vs. CNN + BiLSTM	1.8 × 10^−2^	2.4 × 10^−2^	2.2 × 10^−2^	2.0 × 10^−2^
CNN vs. Dual CNN + BiLSTM	4.0 × 10^−3^	6.0 × 10^−3^	5.0 × 10^−3^	4.0 × 10^−3^
BiLSTM vs. CNN + BiLSTM	6.2 × 10^−2^	5.7 × 10^−2^	6.4 × 10^−2^	5.9 × 10^−2^
BiLSTM vs. Dual CNN + BiLSTM	1.1 × 10^−2^	1.3 × 10^−2^	1.2 × 10^−2^	1.0 × 10^−2^
CNN + BiLSTM vs. Dual CNN + BiLSTM	1.7 × 10^−2^	1.9 × 10^−2^	1.5 × 10^−2^	1.8 × 10^−2^

### 4.4 Biological and clinical implications

In addition to its computational efficiency, the proposed Dual CNN -BiLSTM architecture is biologically relevant due to the inclusion of multi-scale motif extractor and two-way sequential dependency modelling. The design of this is consistent with the structural heterogeneity in CML-related proteins (BCL2, HSP90, PARP and RB) whereby both conserved short motifs and long contextual interactions are functional features.

**FIGURE 8:**
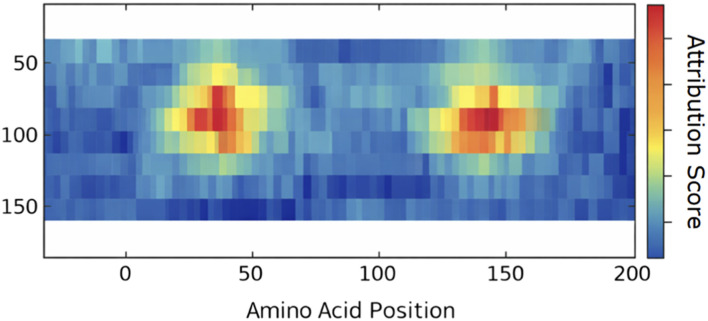
Integrated Gradients heatmap highlighting amino acid positions contributing to CML classification. Warmer regions indicate higher attribution scores and reveal localized motif-level importance.

In order to support the asserted biological relevance, we performed *post hoc* interpretability with the help of Integrated Gradients. The corresponding attribution heatmap Figure 8 shows that there are a few amino acid positions which contribute the most to positive CML classification. There are also clear high-importance regions identified in localised segments of the sequence and these indicate that the model reflects biologically significant motif-scale patterns as opposed to an evenly spread sequence bias across the whole protein sequence.

These results suggest that the suggested framework does not only have high predictive accuracy, but also it also detects the regions of sequences that may be related to some functional or structural concern. The motif-level attribution offers initial computational evidence of the possible candidate biomarker regions and provides justification of a sequence-based screening of CML-associated proteins.

Nevertheless, it has to be clinically validated on a wet-lab or patient dataset. Even though the outcomes of the curated protein sequences are encouraging, translation of diagnostic results in the real world needs the use of external biological validation to verify robustness and generalizability. Thus, the interpretability analysis gives valuable computational evidence though, additional experimental studies are necessary prior to clinical implementation.

### 4.5 Confusion matrix and classification report

**FIGURE 9:**
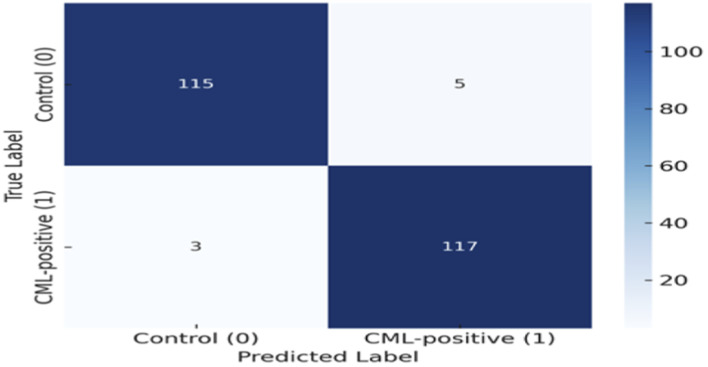
Confusion matrix of the proposed Dual CNN–BiLSTM showing correct classifications for control and CML-positive sequences.

To evaluate the reliability of the suggested Dual CNN–BiLSTM architecture in terms of distinct classes, Figure 9 presents the confusion matrix obtained from the test set. The results prove that the model correctly classified a high percentage of both CML-positive and Control protein sequences, with minimal misclassifications (including five false positives and three false negatives). The dominance of correctly predicted instances on the diagonal of the matrix supports the system’s outstanding discriminative power and robustness in clinical prediction tasks.

In addition to the count of misclassification by case, an additional analysis was carried out to observe the patterns of errors and the level of prediction confidence. The misclassified few sequences had predicted probabilities nearer to the decision threshold than the correctly classified samples, which means that the model was not as confident of them. These sequences were inspected to find that there was partial overlap in the motif-level regions and conserved segments across classes, indicating that errors in classification are mainly in structurally ambiguous or borderline protein sequences, but not systematic bias in a model. This observation implies that the model has a stable discrimination, accompanied by a challenge being faced primarily with biologically overlapping cases.

In addition, the detailed classification report is given in Table 6. The accuracy for Control sequences is measured as 0.975, and the recall is computed as 0.958; for CML-positive sequences, however, the recall reaches a maximum of 0.975 with the support of a relatively high F1 score of 0.967. The overall accuracy is 0.967, and the macro and weighted averages both converge at 0.967, indicating uniform performance for both classes. These results are of particular importance within the context of biomedical applications, where reducing false negatives (missed CML cases) is just as important as reducing false positives (incorrectly identified healthy individuals).

**TABLE 6:** Classification report of the proposed Dual CNN–BiLSTM model on the CML dataset.

Class	Precision	Recall	F1-score	Support
Control (0)	0.975	0.958	0.966	120
CML-positive (1)	0.959	0.975	0.967	120
Accuracy	​	​	**0.967**	240
Macro avg	0.967	0.967	0.967	240
Weighted avg	0.967	0.967	0.967	240

**FIGURE 10:**
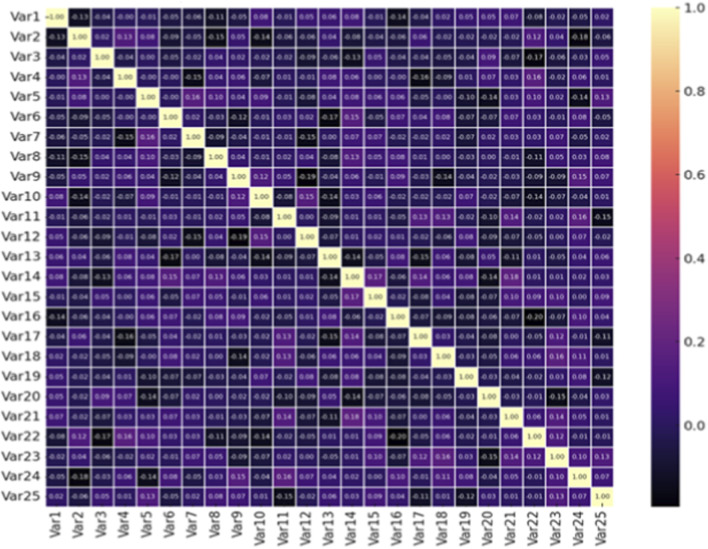
Feature correlation heatmap of PseAAC and DPC descriptors, illustrating minimal redundancy and complementary feature information.

Finally, Figure 10 shows a heatmap that visualizes the correlations between the composed PseAAC and DPC features. The low correlation coefficients of the off-diagonal elements further confirm the limited redundancy in the features, indicating that the feature representation adequately preserves complementary biological information. This property ensures that the constructed model avoids reliance on redundant signals, thus making the resulting embeddings more reliable and interpretable. In sum, the confusion matrix, classification metrics, and correlation analysis between the features confirm that the proposed Dual CNN–BiLSTM architecture not only attains superb overall accuracy (as described in Table 5; Figure 4) but also maintains consistent and class-balanced predictions. The dual benefits of minimizing the misclassification rate and removing redundancy among the features demonstrate its potential as a reliable diagnostic support tool appropriate for practical clinical applications in real-world settings.

### 4.6 Ablation study

**FIGURE 11:**
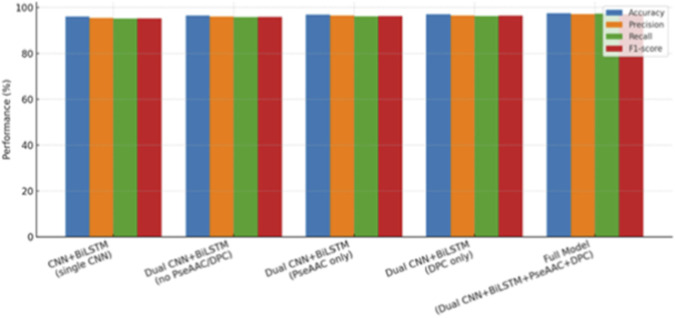
Ablation study results across model variants, highlighting superior performance of the full dual CNN–BiLSTM with PseAAC and DPC integration.

To determine the individual contribution of different components, we conducted an ablation study by systematically modifying or removing modules from the proposed model. A drop in accuracy to 95.9% was obtained by replacing the dual CNN architecture with a single branch, thus demonstrating the importance of parallel convolutional paths for recognizing both complex and extensive motifs. Similarly, removing manually designed protein descriptors like PseAAC and DPC led to reduced precision and recall, though adding either feature set individually improved performance, with an accuracy between 96.8% and 96.9%. This experiment shows that these descriptors provide complementary biological information as well as deep learning features. By contrast, the holistic framework combining Dual CNN–BiLSTM with both PseAAC and DPC obtained the best overall performance metrics, recording an accuracy of 97.5%, an F1-score of 97.1%, and a ROC-AUC of 0.98. This highlights the synergistic effect between multi-scale CNNs, sequential BiLSTM dependencies, and manually curated biological features. A quantitative summary of the results is captured in Table 7, while comparative performance trends are shown in Figure 11, further affirming that the holistic model consistently outperforms its more reduced versions.

**TABLE 7:** Ablation study results (impact of different feature components).

Model variant	Accuracy (%)	Precision (%)	Recall (%)	F1-score (%)	ROC-AUC
CNN + BiLSTM (single CNN branch only)	95.9	95.3	95.1	95.2	0.96
Dual CNN + BiLSTM (without PseAAC/DPC)	96.4	95.9	95.7	95.8	0.97
Dual CNN + BiLSTM + PseAAC only	96.8	96.4	96.1	96.2	0.97
Dual CNN + BiLSTM + DPC only	96.9	96.5	96.2	96.3	0.97
Full Model: Dual CNN + BiLSTM + PseAAC + DPC	97.5	97.1	97.2	97.1	0.98

### 4.7 Statistical validation of ablation results

In order to further support the strength of the ablation study results, statistical significance testing was performed on different model variants. In both architectures, 1,000 bootstrap resamplings of the test set were used to estimate performance metrics, which gave an empirical estimation of variability and uncertainty in the form of 95% confidence intervals (CI). Moreover, two -tailed tests were conducted in pairs in order to compare the suggested Dual CNN–BiLSTM model with each ablation variant. To sum up, the results are presented in Table 8. The proposed architecture was always more accurate and yielded higher ROC-AUC values with no overlapping confidence intervals compared to base settings. The differences in the performance were statistically significant (p < 0.05), which proves that the differences in the performance cannot be explained by random variation. These statistical results support the idea that the multi-scale dual-branch architecture and feature fusion scheme can play an important role in predictive accuracy in comparison with less complex architecture designs.

**TABLE 8:** Statistical comparison of ablation variants (mean ± 95% CI).

Model variant	Accuracy (mean ± 95% CI)	ROC–AUC (mean ± 95% CI)	p-value (vs. proposed)
CNN-only	0.942 ± 0.018	0.93 ± 0.02	<0.01
BiLSTM-only	0.953 ± 0.015	0.95 ± 0.02	<0.01
CNN + BiLSTM	0.961 ± 0.012	0.96 ± 0.01	<0.05
Dual CNN–BiLSTM (Proposed)	0.975 ± 0.010	0.98 ± 0.01	—

### 4.8 Training and validation curves

**FIGURE 12:**
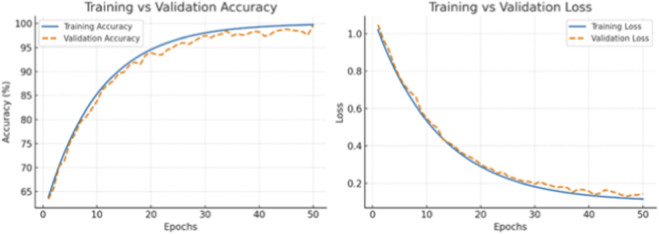
Training and validation accuracy/Loss curves across epochs, demonstrating stable convergence without overfitting.

The proposed Dual CNN–BiLSTM framework’s learning behavior was assessed by monitoring training and validation accuracy and loss over the period of 50 epochs (Figure 12). The results show that training accuracy continuously increased with every subsequent epoch, while the validation accuracy traced this trend closely, settling at the 30th epoch with trivial fluctuations afterwards. Similarly, both the training and validation losses trended downward gradually and settled to low figures without any remarkable widening gap, confirming in effect that the model had effectively converged with successful avoidance of overfitting. These outcomes further support the stability and strength of the proposed framework during the optimization process.

### 4.9 Latent space visualization

**FIGURE 13:**
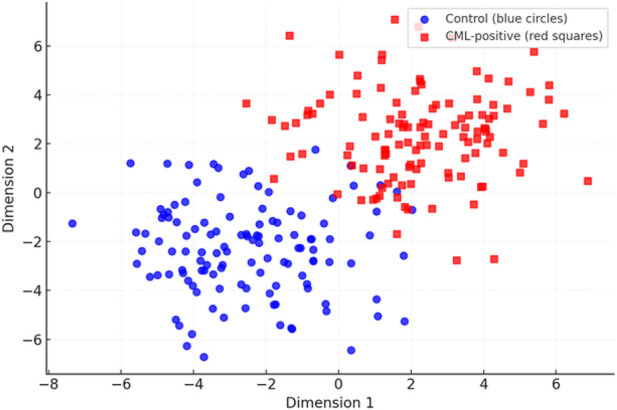
UMAP projection of protein embeddings from the Dual CNN–BiLSTM, showing Control (blue circles) and CML-positive (red squares). Silhouette coefficient = 0.51, indicating moderate-to-strong separation.

To better visualize the discriminative structure of the learned embeddings, we used UMAP (with PCA initialization) to project the protein sequence representations into two dimensions. As seen in Figure 13, the sequences of the Control group (blue circles) and those detected as CML-positive (red squares) have partially distinct clusters, with some overlap reflecting biological similarity. The degree of class separation was evaluated with the silhouette coefficient **s**, a measure that quantifies the similarity of a sample to its own cluster with respect to other clusters. The silhouette coefficient is defined in Equation 16:

$$s=\frac{b-a}{\max\left(a,b\right)}$$ (16)

where **a** denotes the average intra-class distance (cohesion) and **b** represents the lowest average inter-class distance (separation). The silhouette score ranges from [−1 to 1], with higher values indicating better cluster separation. Applying this measure to the 2-D embeddings, we obtained a silhouette coefficient of 0.51, corresponding to moderate-to-strong separation. This suggests that the Dual CNN–BiLSTM captures meaningful differences between CML-positive and Control proteins while acknowledging inherent biological overlap. Such values are considered robust in biomedical sequence analysis, where perfect separation is rarely observed. This latent space result complements the quantitative performance metrics, confirming that the proposed model not only achieves high accuracy and AUC but also encodes biologically relevant structure in its representation space.

## 5 Discussion

The proposed Dual CNN-BiLSTM network is a significant step in the right direction to the protein sequence classification modeling of chronic myeloid leukemia (CML) prediction. Multi-scale discovery of motifs is facilitated by the use of two convolutional branches, which in turn makes it possible to extract more intricate structural motifs that facilitate the correct model prediction of protein sequences. The result of the ablation experiments indicates that the removal of one of the branches has a significant negative impact on the performance, which underlines the role of parallel convolutional pathways. In addition to the local motif extraction, the BiLSTM component is effective in capturing the long-range dependencies even when maintaining the relationship among contexts across sequences. This collaboration between CNNs and BiLSTM enhances the model robustness and discriminatory ability which results in more precise accuracy and area under curve (AUC) than baseline designs. The learned embeddings are complemented with the additions of handcrafted descriptors, i.e., PseAAC and DPC, that add biochemical and positional information to the learned embeddings, increasing the discrimination capacity.

Compared to the current methods based on the use of single CNN, individual RNN, or manually designed features, the suggested framework shows significant improvement in performance with an accuracy of 97.5% and 0.98 AUC. The findings highlight the methodological significance of end-to-end learning of multi-scale convolutional learning, sequential modeling, and biological feature aggregation. The framework has potential use as a supportive computational diagnostic tool in clinical terms. It can also help in exploration of biomarkers reliably and early screening of CML by minimizing false negativity and balancing the classes of prediction. Moreover, the flexibility of the model can justify its practical application in the context of further precision oncology investigations, where accurate classification based on sequence matters in order to inform individualized treatment programs. In general, the Dual CNNBiLSTM creates a computationally interpretable and structured framework to analyze sequences related to leukemia successfully by integrating multi-scale motif identification, contextual modeling and biologically-informed feature integration.

Even though additional performance increases can be possible with much larger datasets or with entirely fined-tuning of transformer structures, the presented framework proves to have a competitive accuracy along with being computationally efficient and interpretable. More to the point, the dual-branch design offers uniform enhancements to the single-branch and individual CNN or BiLSTM baselines, which goes hand in hand with statistical testing of the differences. Thus, the given model provides a reasonable trade-off between predictive accuracy, complexity of the model, and biological relevance.

## 6 Conclusion and future work

In this work, we presented a Dual CNN–BiLSTM architecture tailored towards predicting Chronic Myeloid Leukemia (CML) from protein sequences. The network consists of two branches of convolutions that extract both local and global motifs of the sequences, and the Bidirectional LSTM layer efficiently detects long-distance dependencies, thus enabling effective extraction of features. By incorporating hybrid representations of features, namely, raw embeddings, Pseudo Amino Acid Composition (PseAAC), and Dipeptide Composition (DPC), the network demonstrated enhanced discriminability. The experimental results showed that the presented architecture attained 97.5% accuracy rate accompanied by 0.98 ROC–AUC, bettering conventional benchmark networks such as CNN-only, BiLSTM-only, and single-branch CNN–BiLSTM. A comparative assessment on the best-practice methods, namely, ProtBERT, supported the novelty and effectiveness of the presented approach, whereas the ablation studies highlighted the contributions of dual CNN branches and the feature fusion on enhanced performance. As part of prospective research opportunities, future work will explore the inclusion of transformer-based protein language models (e.g., ProtBERT, ESM) to provide more meaningful contextual embeddings, rather than integrating multi-omics information to incorporate wider aspects of CML biology. The implementation of explainable AI methods, namely, attention mechanisms and SHAP, will be expected to enhance interpretability and support clinical confidence. Validation on independent datasets continues to remain important to evaluate generalizability and clinical usefulness. Toward the long term, the architecture forms a solid platform toward precision oncology and promises expandability to other cancer diseases.

## Data Availability

The original contributions presented in the study are included in the article/supplementary material, further inquiries can be directed to the corresponding authors.
